# Genome Mining Shows Ubiquitous Presence and Extensive Diversity of Toxin-Antitoxin Systems in *Pseudomonas syringae*

**DOI:** 10.3389/fmicb.2021.815911

**Published:** 2022-01-12

**Authors:** Prem P. Kandel, Marina Naumova, Chad Fautt, Ravikumar R. Patel, Lindsay R. Triplett, Kevin L. Hockett

**Affiliations:** ^1^Department of Plant Pathology and Environmental Microbiology, Pennsylvania State University, University Park, PA, United States; ^2^Department of Plant Pathology and Ecology, The Connecticut Agricultural Experiment Station, New Haven, CT, United States; ^3^The Huck Institutes of the Life Sciences, The Pennsylvania State University, University Park, PA, United States

**Keywords:** *Pseudomonas syringae*, toxin-antitoxin systems, phylogroup, genomics, stress

## Abstract

Bacterial toxin-antitoxin (TA) systems consist of two or more adjacent genes, encoding a toxin and an antitoxin. TA systems are implicated in evolutionary and physiological functions including genome maintenance, antibiotics persistence, phage defense, and virulence. Eight classes of TA systems have been described, based on the mechanism of toxin neutralization by the antitoxin. Although studied well in model species of clinical significance, little is known about the TA system abundance and diversity, and their potential roles in stress tolerance and virulence of plant pathogens. In this study, we screened the genomes of 339 strains representing the genetic and lifestyle diversity of the *Pseudomonas syringae* species complex for TA systems. Using bioinformatic search and prediction tools, including SLING, BLAST, HMMER, TADB2.0, and T1TAdb, we show that *P. syringae* strains encode 26 different families of TA systems targeting diverse cellular functions. TA systems in this species are almost exclusively type II. We predicted a median of 15 TA systems per genome, and we identified six type II TA families that are found in more than 80% of strains, while others are more sporadic. The majority of predicted TA genes are chromosomally encoded. Further functional characterization of the predicted TA systems could reveal how these widely prevalent gene modules potentially impact *P. syringae* ecology, virulence, and disease management practices.

## Introduction

The *Pseudomonas syringae* species complex consists of a monophyletic group of plant-pathogenic, plant-commensal, and environmental isolates within the major clade of the genus *Pseudomonas* (Xin et al., 2018). The strains are grouped into 13 phylogroups by multilocus sequence analysis (Berge et al., 2014) and phylogenomics (Gomila et al., 2017; Dillon et al., 2019a; Newberry et al., 2019). Based on host range and symptomatology, *P. syringae* strains have been also classified into over 60 pathovars that together affect almost all known crop plants of economic importance. Periodic outbreaks, including a recent devastating epidemic of bacterial canker in kiwifruit in New Zealand (McCann et al., 2013), establish these plant pathogens as a serious threat to global food production. In addition to being a pathogen, *P. syringae* survives well in the environment and is commonly found in precipitation and natural bodies of water (Morris et al., 2008, 2013). Epiphytic and environmental populations face extreme fluctuations in UV light, temperature, humidity, and nutrient availability, as well as antimicrobial compounds produced by plants and their microbiota (Hirano and Upper, 2000; Lindow and Brandl, 2003; Craig et al., 2021). Previous studies have reported induction of viable but not culturable (VBNC) state and a concomitant alteration in the gene expression profile upon exposure of *P. syringae* cells to conditions that mimic oxidative burst of early stage plant infection (Mock et al., 2015; Postnikova et al., 2015). We recently reported that a plant pathogenic *P. syringae* can survive bacteriocin and antibiotics exposure by entering another dormant, tolerant state known as persistence (Kandel et al., 2020; Patel et al., 2021). Although several stress response pathways have been identified, it is still not well understood how *P. syringae* can cope with environmental stresses in epiphytic and free-living conditions or maintain virulence genes in the extended absence of host selection pressure.

Toxin-antitoxin (TA) systems are small, self-regulating genetic elements, comprised of two to three adjacent genes. The toxin component interferes with an essential cellular function such as translation, DNA replication, and cell wall biosynthesis, whereas the cognate antitoxin neutralizes toxin activity (Unterholzner et al., 2013b). Because the antitoxin is typically less stable than the toxin, conditions that affect antitoxin expression or degradation can increase the pool of unbound toxin, inhibiting cellular processes (Unterholzner et al., 2013b). TA systems have been broadly classified into eight types depending on the nature of antitoxin and its mode of interaction with the toxin (Song and Wood, 2020b). Most well-studied TA systems are Type II, characterized by protein antitoxins that directly bind and neutralize toxin active sites (unlike the type I and III systems, where antitoxins are non-coding RNAs). TA systems were initially implicated as a plasmid maintenance mechanism that suppressed the growth of plasmid-free daughter cells in the absence of antitoxin expression (Ogura and Hiraga, 1983). Subsequent studies have identified TA genes in bacterial chromosomes, which can similarly prevent loss of genomic islands (Wozniak and Waldor, 2009; Yao et al., 2015). TA loci have been implicated in a wide range of roles in addition to plasmid and genome maintenance, including physiological tolerance to antibiotics (Moyed and Bertrand, 1983), phage defense (Sberro et al., 2013; Song and Wood, 2020a), biofilm formation, and virulence (Wood and Wood, 2016). Recent studies have demonstrated that TA systems can impact the ecology, plasmid maintenance, chemical control tolerance, and virulence capabilities of plant pathogens. For example, in *Acidovorax citrulli*, which causes bacterial fruit blotch of cucurbits, expression of a TA operon was significantly induced during plant infection (Shavit et al., 2016). In *Xylella fastidiosa*, overexpression of a TA toxin decreased virulence and increased persistence to copper exposure (Merfa et al., 2016), while in a separate study, deletion of another TA system caused a hypervirulence phenotype suggestive of a regulatory role governing systemic infection (Burbank and Stenger, 2017). In *Xanthomonas oryzae* pv. *oryzicola*, the toxin of one TA system functions as a virulence effector delivered into host cells via the type III secretion system (Triplett et al., 2016). Moreover, in *Erwinia amylovora*, a 10-fold greater tolerance to streptomycin was reported when a TA toxin gene was expressed (Peng et al., 2021). TA systems were also shown to play a significant role in maintenance of *P. syringae* virulence plasmids (Bardaji et al., 2019). While TA systems have been predicted in several plant pathogenic species, including in *Xanthomonas citri* and *Erwinia amylovora* (Martins et al., 2016; Shidore et al., 2019), TA system composition and diversity has not been described in the *P. syringae* species complex.

In this study, we used genome mining to predict TA systems in 339 *P. syrinage* strains representing the broad diversity of the species complex, examined their abundance, diversity, and association with strain phylogeny, source of isolation, and virulence determinants. We show that homologs of well-characterized TA systems are ubiquitous and present in multiple copies in *P. syringae* genomes. We identified six major TA families conserved across species, and others that have more sporadic distributions. Most TA systems occurred in the chromosome and were likely associated to mobile elements and virulence factors. Further experimental characterization of the predicted TA system will reveal their significance in the context of *Pseudomonas syringae* biology, ecology, and virulence, and can provide important insights on how these bacteria can survive in very diverse and harsh environments. These insights could be important in designing new management options for the pathogens.

## Materials and Methods

### Selection of Strains, Phylogenomics, and Phylogroup Designation

We retrieved a set of 2,345 genomes identified as *Pseudomonas syringae* complex in the NCBI database as of October 2020, and generated a preliminary phylogenetic tree using the evolutionary distance estimation method of andi with default settings (Haubold et al., 2014). From this tree, we manually extracted 357 genomes representing the genetic diversity within the *P. syringae* complex. We then assessed the completeness of the assembly and annotation with the benchmarking tool BUSCO (Simão et al., 2015), using the Pseudomonadales single-copy gene ortholog dataset. Genomes with a BUSCO score of less than 95% complete genes were filtered out, and 339 genomes were retained for further analysis (Supplementary Table 1).

A phylogenomic tree of the 339 strains was constructed using GToTree v1.5.46 using Gammaproteobacteria gene profiles (Lee, 2019). Sequences of 172 homologous gene sets were used to construct the tree. The clade containing *P. fuscovaginae* SE-1 was used as an outgroup to root the tree. Clustering of strains with previously known phylogroup designation was used as the basis for assigning phylogroups to unknown strains, after confirming that strains of the same phylogroup clustered together. Together, we included 77, 104, 38, 31, 11, 2, 61, 2, 4, 4, 2 strains from phylogroups 1, 2, 3, 4, 5, 6, 7, 9, 10, 11, and 13, respectively. The *P. fuscovaginae* group clade was not assigned to any phylogroup. Our genome list did not have any representatives from phylogroups 8 and 12 due to unavailability of quality genomes in the public database.

### Prediction of Toxin-Antitoxin Systems

Type II and IV TA systems were predicted for whole genomes of all 339 strains using SLING 2.0.1 (Horesh et al., 2018), a program that identifies gene pairs based on Hidden Markov Models (HMM) alignments to annotated genes or unannotated putative ORFs, as well as gene pair structural requirements. Nucleotide sequence, annotations, and toxin profile HMMs included with the program were used as inputs with default parameters. SLING was run on Roar high-performance computing platform using 16 processors at Penn State. SLING output consisted of 260 hits that contained a gene with a known toxin domain (Supplementary Figure 1). A hit is a CDS with a HMMER bit score of at least 20 for overall sequence/profile comparison with a previously characterized and/or predicted toxin CDS included in the program. Hits with the same toxin gene domain were given separate designations using an *e*-value cutoff of 0.01 and an identity score of 30% as reported previously (Horesh et al., 2018). Toxin hits sharing common domain names were considered homologs and combined into one category to simplify the results. For example, the *parE* toxin gene included 47 hits that were combined to form a single ParDE TA system. The homologs with the same hit label (eg., 4H-*parE*) across the strains (i.e., orthologs) were highly conserved (> 70% amino acid identity) in their amino acid sequence, while hits within a strain (i.e., paralogs) with different labels (eg., 4H-*parE* and 11H-*parE*) were divergent (< 30% identity) (Supplementary Figure 2). To check for completeness of the SLING prediction, we acquired the TA systems of the three model strains (DC3000, B728a, and 1448A) predicted in TADB2.0, an updated database of bacterial type II TA loci (Xie et al., 2018). The TAFinder tool of TADB2.0 was also run with default parameters using the Refseq accession numbers of the model strains.

To predict TA systems not included in the training sets of above programs, an HMM alignment approach was used to screen genomes for type I, III, V, and VI systems. For each family of toxin and protein antitoxin to be searched, a minimum of 20 amino acid sequences representing that family across diverse bacterial species were retrieved from UniProt. Sequences were aligned with MAFFT v 7.450 using default settings and an HMM profile was built using default settings of the hmmbuild function in the HMMER webserver (Finn et al., 2011). A locally created target database of 339 *P. syringae* genomes translated into six reading frames was used in HMMER’s HMMsearch. The TA systems screened this way were type I (*hok*/*sok*, *ldrD*/*rdlD*, *tisB*/*istR-1*, *symE*/*symR*, *ibsC*/*sibC*, *txpA*/*ratA*, *fst*/*RNAI*-*RNAII*, *pndA*/*pndB*, *shoB*/*ohsC*); type III (*toxN*/*toxI*, *abiQ*/*antiQ*, *cptI*/*cptN*, *tenpN*/*tenpI*); type V (*ghoT*/*ghoS*); and type VI (*socB*/*socA*). A type I TA system predicted for strain DC3000 in the type I toxin-antitoxin database T1TAdb (Tourasse and Darfeuille, 2021) was included in the analysis after confirming its presence in our genomes by nucleotide and protein BLAST.

Heatmaps were created using the Interactive Tree of Life (iTOL) v5 (Letunic and Bork, 2021) using the phylogenomic tree and TA system abundance used as annotation. Predicted TA systems were categorized based on their frequency among strains, as prevalent (if at least one homolog of the given toxin domain is present in ≥ 80% of strains), common (present in 20–79% of strains), or rare (present in < 20% of the strains).

### Association of Toxin-Antitoxin System Abundance With Phylogroup, Genome Size, Plasmid Content, Source of Isolation, and Presence of Virulence Determinants

Differences between phylogroups in TA system abundance were determined from the SLING output and uniquely predicted TA system of TADB2.0 (*psyrTA*), and T1TAdb (*hok-sok*). Genome size was defined as the assembly size and was recorded for all strains, while plasmid counts were based on analysis of 28 closed genomes with resolved plasmids. Chromosome or plasmid localization of genes was determined by manually examining the start and end nucleotide positions of predicted toxin genes. Isolation sources were obtained from strain information within NCBI annotation files, with all isolates from non-plant sources classified as “other” (Supplementary Table 1). To identify correlations between TA system abundance and the abundance of predicted type III effectors, we used bioinformatically predicted effector repertoires of 140 of the 339 of our genomes as computed in a previous study (Dillon et al., 2019b). Putative secretion signals in toxin and antitoxin genes of *P. syringae* strain DC3000 was predicted using EffectiveDB (Eichinger et al., 2016).

### GC Content, Codon Usage Variation, Gene Neighborhood, and Strain Grouping by Toxin-Antitoxin Abundance

To identify signs of TA system acquisition through horizontal transfer, GC content of top 20 abundant toxin hits was extracted and was compared to that of the genomic GC content. Codon usage was extracted from the Codon Usage Database.^1^ The gene neighborhood of the most ubiquitous three TA system homologs (*pasTI*, *RES-Xre*, and *vapBC*) for the three strains DC3000, B728a, and 1448A was visualized using gene graphics.^2^ Grouping of strains based on similar TA system content was performed using SLING outputs of the toxin hits using the average linkage method for clustering and the Euclidean method for distance measurement in heatmapper (Babicki et al., 2016).

### Sequence Search, Blast, Alignment, and Phylogenetics

Sequences of the orthologs and paralogs of the same toxin hits (e.g., *parE* had 47 total homologs with up to 12 paralogs predicted in a single genome) were retrieved from the model strains DC3000, B728a, and 1448A. Alignment of the sequences was performed in Muscle 3.8.425 within Geneious using 10 maximum iterations. A phylogenetic tree of the homologs was constructed using FastTree 2.1.11 using default parameters.

### Literature Search for Toxin-Antitoxin System Expression Patterns

We identified 10 papers for which RNA-Seq or microarray data were reported for strains DC3000, B728a, and 1448A under environmental or mutant conditions. Fold change or log_2_ fold change data was obtained from supplementary materials, and locus IDs corresponding to SLING-predicted TA systems were manually extracted using the start and end nucleotide positions of TA genes in the genome. Significantly differentially expressed loci were reported here as reported by the authors; no new data analysis was performed. Moreover, we used data from a recent study on genome-wide identification of fitness conferring genes in strain B728a and analyzed the fitness profiles of TA genes from their supplementary data (Helmann et al., 2019).

### Statistical Analyses

Statistical analysis of difference in TA counts by phylogroup was performed in JMP Pro 15 using the non-parametric Kruskal-Wallis test *(P* < 0.05) followed by each pair multiple comparisons after confirming that assumptions of parametric tests are not satisfied as per the goodness of fit test and unequal variance test in JMP. Phylogroups having fewer than 10 strains were excluded from this analysis. Correlation analysis between the TA counts and genome size, plasmid content, and type III effector count was also performed in JMP Pro 15 using the multivariate option and Spearman’s ρ. GC content differences were assessed using Kruskal-Wallis test with a genomic GC used as control group in Steel method.

## Results

### *P. syringae* Genomes Encode 26 Toxin-Antitoxin System Families Targeting Diverse Cellular Functions, of Which Six Are Prevalent Throughout the Species

**FIGURE 1:**
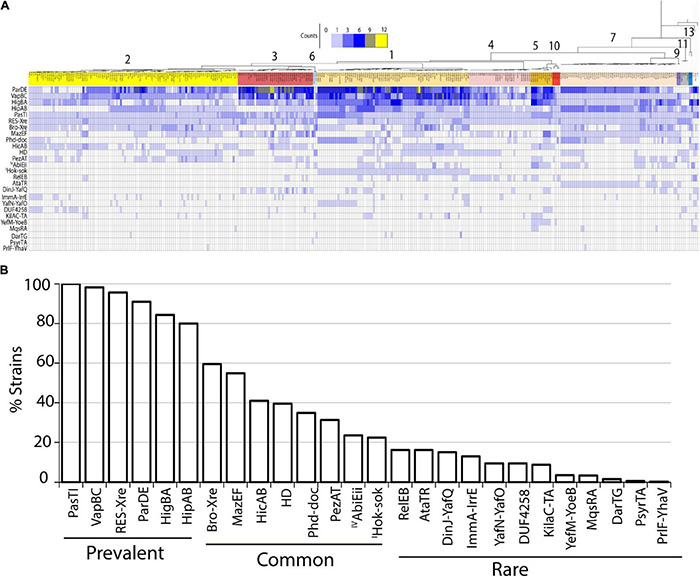
**(A)** Heatmap showing TA system content and distribution across *P. syringae* species complex. Names of TA systems are indicated for each row. Number of homologs of each TA systems are indicated by the color key at the top. Phylogenetic tree color coded according to phylogroups are indicated by numerals. **(B)** Percentage of strains containing the TA systems. List of all the TA systems found in *P. syringae* are shown in the x-axis, and the fraction of strains carrying a given TA system out of the 339 strains is shown in the y-axis. TA systems were categorized as prevalent, common, and rare based on their abundance as shown. Except *abiEii* (type IV), and *hok-sok* (type I) all belong to type II TA families.

As a basic step in understanding the roles of TA systems in *P. syringae* ecology, we aimed to obtain a comprehensive list and abundance patterns of TA systems across the species complex using bioinformatics prediction tools including SLING, BLAST, and HMMER. We predicted 26 distinct TA system families throughout the *P. syringae* genomospecies (Figure 1 and Supplementary Figure 3). Of these, 24 belonged to the type II TA system families, and one each belonged to type I (*hok-sok*) and type IV system (a*biEii*) families. The majority of TA system toxins predicted are orthologs of toxins in other species, which have been previously reported to inhibit translation through mRNA cleavage or through modification of translation factors, while others are reported to inhibit replication, cell wall biosynthesis, NAD + homeostasis, and other essential functions (Table 1). To confirm the comprehensiveness of the SLING method, predictions from three *P. syringae* model strains were compared to that from the database TADB2.0. The majority of the SLING predictions were also identified in TADB2.0 (7 of 12 in B728a, 14 of 19 in DC3000, and 1448A), including five of the six most prevalent systems described below. Six TA systems, including the highly conserved *pasTI* module, were only predicted using SLING. A TA system from a validated family, *psyrTA* (Sberro et al., 2013), was predicted in TADB2.0 but not SLING. Few TA genes were uniquely predicted in TADB2.0 and TAFinder (Supplementary Table 2).

**TABLE 1:** Toxin-antitoxin systems predicted in *P. syringae*, toxin function and physiological effect.

TA system	Toxin domains	Toxin function	Function affected	References
ParDE^P^	ParE	DNA Gyrase inhibitor	Replication	Jiang et al., 2002
VapBC^P^	PIN, PIN_3, DUF4411	Cleavage of tRNA^fMet^	Translation	Winther and Gerdes, 2011
HigBA^P^	HigB, HigB-like, Gp49	50S ribosome dependent mRNA cleavage	Translation	Hurley and Woychik, 2009
HipAB^P^	HipA_C, HipA_C-HipA_N-Couple_hipA	Phosphorylation of glutamyl tRNA synthase (GltX)	Translation	Germain et al., 2013
PasTI^P^ (YfjG-YfjF)	RatA, polyketide_cyc2	Binds 50S ribosomal subunit and blocks 70S ribosome formation	Translation	Zhang and Inouye, 2011
RES-*xre*^P^	RES	ADP ribosylates phosphoribosyl pyrophosphate synthetase (Prs)	NAD + homeostasis	Piscotta et al., 2019
Bro-TA^C^*	Bro-N	?	?	
MazEF^C^	PemK	Ribosome independent mRNA and rRNA cleavage	Translation	Zhang et al., 2003
Phd-doc^C^	Fic, DUF4172	Phosphorylates elongation factor Tu (EF-TU)	Translation	Cruz et al., 2014
HicAB^C^	HicA	Ribosome independent mRNA cleavage	Translation	Jørgensen et al., 2009
HD-TA^C^	HD	c-di-AMP hydrolysis	Signaling?	Huynh et al., 2015
PezAT^C^ (Epsilon-Zeta)	Zeta	Phosphorylate uridine diphosphate-N-acetylglucosamine (UNAG)	Cell wall biosynthesis	Mutschler et al., 2011
AbiEii^C^ (MenTA)	AbiEii NTP_transf_2	Nucleotidyltransferase on serine tRNA	Translation	Cai et al., 2020
Hok-sok^C^	Hok	Membrane depolarization	Membrane	Gerdes et al., 1986
RelEB^R^	RelE_like	Ribosome bound mRNA cleavage	Translation	Christensen and Gerdes, 2003
AtaTR^R^	GNAT_acetyltran	Transfers an acetyl group from acetyl coenzyme A to Met-tRNAfMet.	Translation	Jurënas et al., 2017
DinJ-YafQ^R^	YafQ	Ribosome dependent mRNA cleavage	Translation	Prysak et al., 2009
ImmA-IrrE^R^	Peptidase_M78, DUF955	Metalloprotease, activation causes transcriptional induction of genes required for repair and survival after radiation exposure	Cleaves repressor, and induces transcription	Ludanyi et al., 2014
YafN-YafO^R^	YafO	Ribosome dependent mRNA cleavage	Translation	Zhang et al., 2009
DUF4258^R^	DUF4258, CdiA	DNA double strand break (contact dependent inhibition protein)	DNA damage	Roussin et al., 2019
KilAC-TA^R^*	ANT	? (Could be involved in phage repression)	?	
YefM-YoeB^R^	YoeB	50S ribosomal subunit dependent mRNA cleavage at A site	Translation	Zhang and Inouye, 2009
MqsRA^R^	MqsR	Ribosome independent mRNA cleavage	Translation	Yamaguchi et al., 2009
DarTG^R^	DUF4433	ADP ribosylates DNA	Replication	Jankevicius et al., 2016
PsyrTA^R^	RecQ	ATP-dependent DNA helicase	?	Sberro et al., 2013
PrlF-YhaV^R^	Toxin_YhaV	Ribosome dependent mRNA cleavage	Translation	Choi et al., 2017

*These systems were predicted by bioinformatics analysis. Overexpression was toxic to cell, but TA function was not confirmed (Chan et al., 2014). ^P^prevalent, ^C^common, ^R^rare.

Genomes varied substantially in TA system repertoires, and only six TA system families were highly prevalent (i.e., present in ≥ 80% of the genomes analyzed (Figures 1A,B). These included systems encoding putative translation inhibitor toxins (*pasTI*, *vapBC*, *hipAB*, and *higBA*), a replication inhibitor (*parDE*), and a RES domain toxin proposed to interfere with cellular NAD^+^ homeostasis (Table 1). Eight additional predicted systems present in more than 20% of the strains were categorized as common TA systems, and the remaining 12 were rare (Figure 1B). Prevalent TA system families included sets of toxin orthologs that diverged across strains despite sharing the common toxin domain, and some included multiple distinct paralogs within a genome. For example, for the *parE* toxin gene of the *parDE* system, 47 variants were predicted (Supplementary Figure 1). Similarly, the *vapBC* family had 21 distinct variants. A conserved copy of *pasTI*, *RES-xre*, and *vapBC* systems were predicted in all or most genomes (100, 94, and 87%, respectively), suggesting an important conserved function.

A *hok-sok* module of type I TA system was predicted in the strain DC3000 in the T1TAdb database (Tourasse and Darfeuille, 2021). Nucleotide BLAST of the predicted sequence in the locally constructed database of our 339 genomes confirmed presence of full complement of this system in strains of phylogroup 1 (Figure 1A). Other genomes contained orthologs of only the toxin component. Alignment of the predicted Hok sequence to previously characterized Hok homologs showed low identity (15% to that of pR1 plasmid and 13% to that of *Erwinia amylovora*). No TA toxin genes for any of the type I, III, V, and VI systems were detected using BLAST and HMMER alignment methods. All strains contained a homolog to the proposed type IV TA system *cptB/sdhE* antitoxin that is encoded in an operon with a hypothetical protein of unknown function (DC3000 locus ID: PSPTO_4227), but no *cptA* toxin was identified, and *sdhE* is an essential flavinylation factor in bacteria, so this is not a likely TA system. A few potential homologs were detected for antitoxins *abiGii* (type IV), *ghoS* (type V), and *socA* (type VI). Since these did not occur in the type strains and no toxin homolog was detected, no further assessment was performed.

### Toxin-Antitoxin System Abundance Differs by Phylogroup of *P. syringae*

**FIGURE 2:**
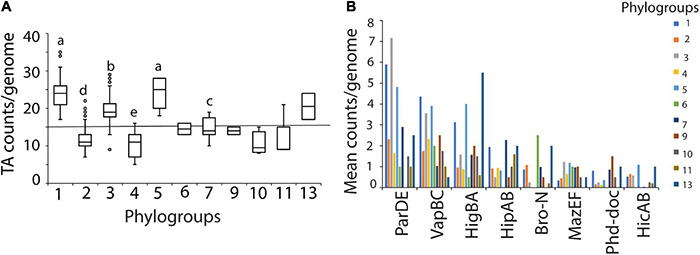
**(A)** TA counts per genome by phylogroups. The central line shows median number of TA system genes from the 339 strains used. Different letters indicate significant difference in counts as analyzed by Kruskal-Wallis test at *P* < 0.05. Phylogroups with less than 10 strains were not used in the analysis. **(B)** Differences in major TA system content among phylogroups. Mean counts per genome by phylogroup are shown. Phylogroups differed in copy number per genome of *parDE*, *vapBC* and *higBA* families (also refer heatmap in Figure 1A).

As TA system abundance greatly varied across genomes with a minimum of 5 to a maximum of 35 TA systems (median of 15) predicted per genome of *P. syringae*, we aimed to understand the determinants that influence TA system abundance. TA system abundance varied significantly across phylogroups (Figure 2A). Phylogroups 1, 3, and 5 had significantly more TA systems per genome than other phylogroups (Figure 2A). These differences were mainly driven by a large variation in the within-genome abundance of three of the prevalent TA systems, *parDE*, *vapBC*, and *higBA* (Figure 2B). In addition to the copy number differences, phylogroups also differed in composition of TA system families. For example, putative *bro-xre* systems were common in phylogroup 2, but absent in phylogroups 4, 5, and 10. The *mazEF* system was ubiquitously predicted in phylogroup 7 strains but was sporadic in others (Figures 1A, 2B). Similarly, full complement of the type I *hok-sok* system was predicted only in phylogroup 1 (Figure 1A). Phylogroups could also be distinguished by divergent orthologs of the same families; for example, the HipA-5H homologs were predominantly found in phylogroups 7, while the HipA-6H group was concentrated in phylogroups 1 and 4 (Supplementary Figure 1).

In a clustering analysis based on TA system repertoires, TA system composition followed phylogroup structure in *P. syringae*, with >90% of strains clustering with their phylogroup, suggesting that most TA system acquisition happened prior to phylogroup diversification (Supplementary Figure 4). However, at least 26 strains clustered outside their phylogroups, and phylogroups 1, 4, and 5 were significantly split into two or more clusters, potentially indicative of more recent TA system gain or loss in these strains. Overall, genetic similarity (i.e., phylogroup association) is a major factor governing similarities in TA system abundance.

### Toxin-Antitoxin System Abundance Is Positively Linked to Genome Size and Plasmid Number, and Most Toxin-Antitoxin Systems Are Chromosomally Encoded

**FIGURE 3:**
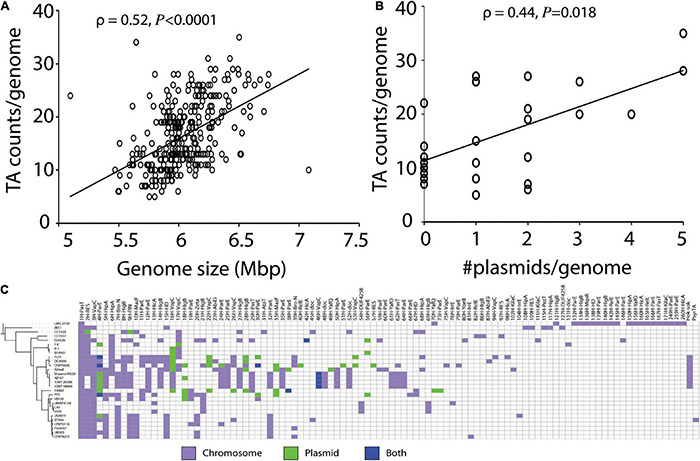
**(A)** Association of TA counts by genome size and **(B)** plasmid counts. **(C)** Heatmap showing occurrence of all 260 homologs of TA systems in the 28 completely sequenced strains in chromosome or plasmids. Most TA systems were encoded in the chromosome.

TA systems frequently occur in mobile genetic elements (MGE), that are known to be horizontally transferred. As the genome sizes increase due to integration of MGEs or presence of plasmids, we hypothesize that the number of TA systems will also increase. We performed a correlation analysis on 339 strains to determine whether predicted TA system abundance is associated with genome size. Genome size was weakly, though significantly, correlated to TA system count (ρ = 0.52, *P* < 0.0001, Figure 3A). There was a similar positive correlation observed between TA system count and the number of plasmids (ρ = 0.44, *P* = 0.018, Figure 3B).

Next, we examined whether the TA systems predicted in the 28 complete *P. syringae* genomes were chromosome- or plasmid-borne. The majority (45–100%, mean = 87.7 ± 14.3) of predicted TA systems were encoded in the chromosome (Figure 3C and Supplementary Figure 3). The most widely distributed TA systems, with *pasT*, *vapC*, and RES domain toxins, were exclusively or almost exclusively detected on chromosomes, though the common and rare *vapC* homologs also occurred in plasmids (Figure 3C). Of the 40 plasmids analyzed, 33 contained at least one TA system with a mean count of 1.8 TA systems per plasmid, and 7 plasmids did not contain TA systems. A maximum of eight TA systems were predicted in the large plasmid of the strain 1448A.

We next analyzed GC content, codon usage variation, and genomic context of TA genes to detect if they contain signs of horizontal transfer. By comparing the GC content of top 20 toxin hits to that of the genome, we showed that GC content of 17 of the 20 toxin genes significantly differed from that of the genome. GC content varied by > 5% in 8 of the 20, and by > 3% in 12 of the 20 toxin genes (Supplementary Figure 5). Although we did not detect a noticeable codon usage variation compared to the genome in strain DC3000, few toxin genes such as *parE*, *hicA* and *higB* showed minor differences (Supplementary Figure 6). Upon examining the genomic context of the three most abundant and highly conserved TA system variants (i.e., *pasT*, RES, and *vapC*), we observed that these systems are immediately flanked by or lie within 20 kb region from MGE in at least one of the strains (Supplementary Figure 7). Together, these results suggest that most TA systems in *P. syringae* occur in mobile elements and are likely to be acquired by horizontal transfer.

### Isolation Source of Strains Does Not Affect Toxin-Antitoxin System Abundance, and Toxin-Antitoxin Gene Counts Are Weakly Correlated to Type III Effector Gene Counts

**FIGURE 4:**
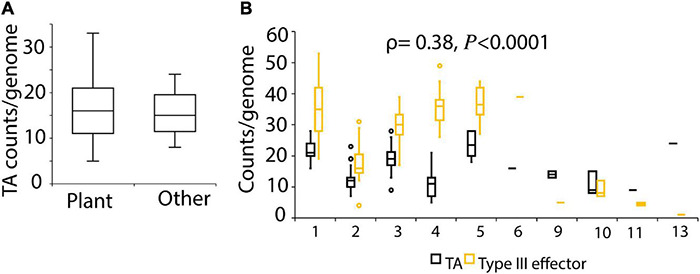
**(A)** TA counts by isolation source of strains. Strains isolated outside of plant environment (rain, snow, stream, lake water, epilithon) were categorized as other (*n* = 13). **(B)** TA counts and type III effector counts by phylogroups (x-axis). Type III secretion system effector counts predicted in 140 genomes from a previous study (Dillon et al., 2019b) were used. Strains of phylogroup 7 were not used in this analysis as the strains used here were not used in Dillon et al. (2019b).

A previous study reported higher abundance of TA systems in free-living than in host-associated isolates (Pandey and Gerdes, 2005). We therefore analyzed if the source of isolation of the strains influences the abundance of TA system in *P. syringae.* We did not detect any difference in TA abundance based on the environment from which strains were isolated (Figure 4A and Supplementary Figure 8). Notably, strain Psy642, which forms a separate subclade 2c within phylogroup 2, lacks the major virulence determinant of canonical type III secretion system, and was reported to be avirulent in a previous study (Demba Diallo et al., 2012), showed a different pattern of TA content than other strains of the same phylogroup (Supplementary Figure 1). Psy642 and two other strains in this clade did not contain the *vapBC* genes (Figure 1A). The highly prevalent *vapC*-3H, *parE*-4H, and *hipA*-5H homologs are missing in these strains (Supplementary Figure 1). Since TA systems are known to stabilize genomic islands that contain virulence and antibiotics resistant determinants (Wozniak and Waldor, 2009; Yao et al., 2015), we hypothesized that the predicted type III secretion system effector counts are correlated to TA gene counts. To this end, we examined the association between the number of TA loci and type III effectors, as predicted in a previous study (Dillon et al., 2019b). Although a similar trend was observed in the TA gene counts and type III effectors in the three phylogroups (1, 2, and 3) that are dominated by plant pathogens, overall, a weak positive correlation (ρ = 0.38, *P* < 0.0001) was detected between the two variables (Figure 4B). We also attempted to examine the association of TA genes to markers of copper and streptomycin resistance, bactericides that are frequently used in control of *P. syringae* diseases. However, these markers could be predicted only in a limited number of strains based on BLAST search of the resistant genes as query against our local database of 339 genomes.

EffectiveT3 predicted four TA gene products of DC3000 as type III secreted proteins. These included antitoxins of *vapBC*-3H (PSPTO_2000), *vapBC*-26H (PSPTO_1058), and toxins of HD-15H (PSPTO_2479), and *ataTR* (PSPTO_RS175570).

### Published Gene Expression and Fitness Profiles for Predicted Toxin-Antitoxin Systems Indicate Distinct Regulatory Patterns

Although TA systems are posttranslationally regulated, some antitoxins are repressors of the TA operon, and conditions leading to antitoxin degradation and excess free toxin might lead to induction of the TA operon (Chan et al., 2016). Therefore, expression patterns could point to conditions in which certain toxins might be functional. We compiled reported transcriptional differences from nine RNA-seq studies and one well-replicated microarray study, all performed on one of the model strains DC3000, B728a, or 1448A. Seven of the studies reported significant differential expression of one or more of the four highly conserved TA system genes (*pasTI, RES-xre, vapBC, and parDE)* under host or abiotic stress, or in virulence regulatory mutants (Table 2), while three studies found no difference. In microarray analysis of strain B728a, epiphytic growth and nitrogen starvation induced 3 TA systems. *vapC* toxin genes were reported to be substantially induced during nitrogen starvation (Supplementary Table 3). The expression of *RES-Xre* was suppressed during early infection of DC3000 on *Arabidopsis* plants and was conditionally affected by several regulatory gene deletions in 1448A (Table 2). While these expression patterns do not provide conclusive evidence of a function or lack thereof, they indicate that the different TA system families are regulated in distinct and complex pathogen and environmental contexts.

**TABLE 2:** Reported expression patterns of TA system genes in three model strains B728a, 1448A, DC3000.

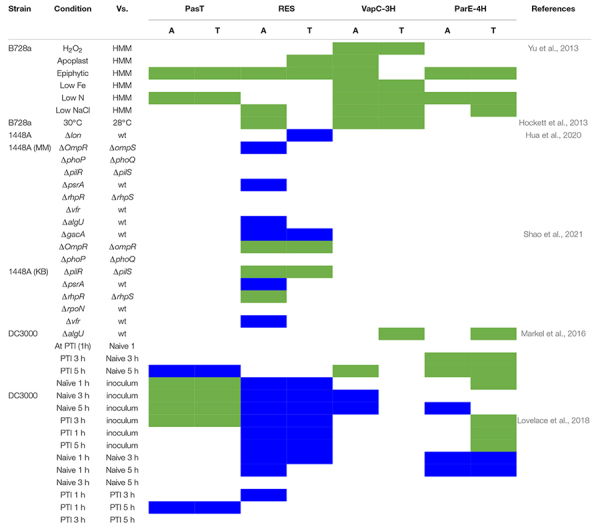

*Green box indicate significantly upregulated gene, blue box indicate the significantly downregulated gene and white box indicate no significant difference in the expression level in comparison with control.*

*Bronstein et al. (2008), (DC3000): RES Antitoxin, down regulated and ParE-1 Toxin, upregulated due to ferric citrate and sodium citrate treatment at 4 h compared to control respectively.*

*Lam et al. (2014), (DC3000)- hrpRS and HrpL mutants: no effect on any of these four TA systems.*

*Filiatrault et al. (2010), (DC3000): no effect on any of these four TA systems.*

*Santamaría-Hernando et al. (2020)20, (DC3000): no effect on TA systems of blue-light perception.*

*Greenwald et al. (2012), (B728a)-B728a ΔacsS vs. B728a: no effect on any of these four TA systems.*

*Liu et al. (2020), (DC3000, B728a) DC3000 Δ(p)ppGpp vs. DC3000 and B728a Δ(p)ppGpp vs. B728a: None of these TA systems are differentially expressed.*

*A, antitoxin; T, toxin; HMM, HRP MM-mannitol medium; Δlon, Lon knockout mutant; ΔOmpR, ΔompS, ΔphoP, ΔphoQ, ΔpilR, ΔpilS, ΔpsrA, ΔrhpR, ΔrhpS, Δvfr, ΔalgU, ΔgacA, and ΔrpoN, deletion mutant of Type 3 secretion system regulators; MM, M9 minimum medium; KB, King’s B medium; PTI, pattern-triggered immunity; 1 h, 1-h post inoculation; 3 h, 3-h post inoculation; 5 h, 5-h post inoculation; hrpRS^–^ and hrpL^–^, deletion mutant of hrpRS and hrpL gene; and DC3000 Δ(p)ppGpp and B728a Δ(p)ppGpp, ppGpp gene knockout mutant of DC3000 and B728a, respectively.*

In a recent Rb-TnSeq study aimed at identifying genome-wide fitness profile of B728a genes, three antitoxin gene mutants (that of two *parDE* and a *vapBC*) showed below threshold abundance in the BarSeq library (Supplementary Figure 9). The authors suggested that these genes could be important for *in vitro* growth and survival. Similarly, although not reported to be significant in the paper, the plant fitness score was lower for antitoxin mutants of *RES-xre*, *hipAB*, *psyrTA*, and a *vapBC* system (Supplementary Figure 9). We speculate that the disruption of antitoxin genes by transposon insertion could have freed their cognate toxins. The freed toxins subsequently caused growth inhibition or death of the mutants lowering their abundance in the library. Interestingly, toxin gene mutants of the *pasTI* system showed below threshold abundance in the library, which could suggest a different regulation of this TA system or multiple cellular functions of the predicted toxin gene. Moreover, in the study that validated TA system function of *psyrTA* mentioned above, a *parDE* system is reported to be functional based on cloning success of predicted TA genes during Sanger-based whole-genome shotgun sequencing (Sberro et al., 2013).

## Discussion

Using *in silico* prediction from a broad-scale genomic data, we show that TA systems are prevalent and occur in diverse patterns in *Pseudomonas syringae* species complex. We found that 100% of screened genomes contain at least five TA systems with a median count of 15 per genome across the species. We predicted 26 distinct TA families that almost exclusively belong to the type II group, except for a type IV system that was sporadically detected in some strains and a type I system detected in phylogroup 1. Of the 26 TA systems, six are highly abundant across the species implying potentially conserved roles in *P. syringae* ecology. The most prevalent among the TA systems was that of the *pasTI* system, occurring mostly as a single copy in all strains with a high degree of sequence conservation. Although limited data exists regarding the functional characterization, a previous study in *Escherichia coli* CFT073 showed that this TA system is involved in stress tolerance under nutrient limitation, oxidative stress, and antibiotic persistence (Norton and Mulvey, 2012). Another recent study has confirmed its role in stress survival but contradicted its function as a TA system (Fino et al., 2020). Interestingly, The *pasT* gene was recently shown to be important for *in vitro* fitness in *P. syringae*, indicating a functional role (Helmann et al., 2019).

Orthologs of other prevalent TA loci (*vapBC*, *RES-xre*, *parDE*, *higBA*, *hipAB*) have been widely described to function as TA systems. We predicted multiple paralogs of *parDE*, *vapBC*, and *higBA* systems in some phylogroups and strains. We determined that sequences of paralogs are highly divergent. Whether all paralogs are active and if they are also functionally diverse is a subject of further research. The *parDE* and *vapBC* homologs were also among the most prevalent in another plant pathogen, *Erwinia amylovora* (Shidore et al., 2019). A *vapBC* ortholog was characterized as a TA system in the plant pathogen *Acidovorax citruli*, and was shown to be overexpressed during plant infection (Shavit et al., 2016). We predicted that some of the *vapB* antitoxin sequences contain a type III secretion signal, indicating a potential role in virulence. The role of *vapBC* in stress responses is reported in several other species (Arcus et al., 2010). The *parDE* system has been implicated in persistence against DNA gyrase inhibiting antibiotics in *P. aeruginosa* (Muthuramalingam et al., 2019). Homologs are also known for plasmid and genome maintenance in several other species including plant pathogens (Roberts et al., 1994; Yuan et al., 2011; Unterholzner et al., 2013a). Similarly, TA systems containing the RES domain toxin was also prevalent, and a copy was highly conserved across species. Although this system is previously characterized as a bona fide TA system including in *P. putida* (Skjerning et al., 2019), its physiological role remains to be determined. *higBA* system, another prevalent TA system in *P. syringae*, was shown to regulate virulence factor production in *P. aeruginosa* (Wood and Wood, 2016). Similarly, *hipAB* system has been shown to function in antibiotics persistence *in vitro* (Moyed and Bertrand, 1983; Germain et al., 2013) and also in the natural host environment (Schumacher et al., 2015). Taken together, the prevelance of these TA systems in *P. syringae* and their characterized roles in other taxa indicate that they may play multiple roles in the ecology of this phytopathogen. Further experiments will be able to reveal more specific details on their roles and the conditions under which they are activated.

TA genes that were less prevelant but were commonly found in *P. syringae* included toxin families of well characterized TA systems in other species. Some notable examples in this category were *mazEF*, *hicAB*, *phd-doc*, *pezAT*, *abiEii*, and *hok-sok*. Previous studies have reported stress and virulence associated function of these TA genes. For example, MazF toxin of the *mazEF* system was shown to contribute *Mycobacterium tuberculosis* cells survive oxidative stress, nutrient depletion, and virulence in the host (Tiwari et al., 2015). Rare TA systems also contained TA candidates that have been experimentally verified in other systems, including toxin families RelE, YafO, YafQ, AtaT (GNAT), YoeB, MqsR, and YhaV. Some candidates of both common and rare TA systems predicted in this study are less well studied and have not been demonstrated to function as TA systems experimentally, such as the Bro-N and KilaC(ANT) toxins (Chan et al., 2014). Only further experimental research can validate if they function as a TA system in *P. syringae* or are involved in other functions.

Our results show that TA system abundance differs by phylogroups. In addition to the genetic similarity, strains belonging to the same phylogroup are generally expected to have similar phenotypic traits consistent with their unique ecology. For instance, phylogroup 2 strains were shown to be more aggressive in cantaloupe seedling, were most consistently ice-nucleation active, and more likely to produce syringomycin-like toxin than strains of other phylogroups. Similarly, phylogroup 3 strains are rarely known to possess ice-nucleation activity and are negative for syringomycin-like toxins (Berge et al., 2014). Previous studies have also reported phylogroup dependent differences in the content of virulence factors (Baltrus et al., 2012; Dillon et al., 2019b). While it is impossible to speculate the significance of these difference at this point, similar sporadic distribution of TA system linked to genetic similarity has been also described previously both in clinical (Horesh et al., 2020) and plant pathogens (Shavit et al., 2016; Shidore et al., 2019).

TA systems are mostly known to occur in the accessory genome and are associated to MGEs or plasmids (Fraikin et al., 2020). Although we predicted that 82.5% (33 out of 40) of plasmids contain at least one TA system, a vast majority of TA systems are encoded in the chromosome in *P. syringae*. Chromosomally encoded TA systems are also reported to contribute to stability of genomic islands (Wozniak and Waldor, 2009; Yao et al., 2015). Moreover, virulence and antimicrobial resistance determinants are known to occur in laterally acquired genomic islands including in *P. syringae* (Kim et al., 1998; Feil et al., 2005). A comparison of the counts of type III effectors and TA loci showed similar trends in three of the phylogroups that are dominated by plant pathogens. Phylogroup 2 strains, which are known to be broad-host range and contain relatively smaller repertoire of type III effectors compared to other phylogroups (Baltrus et al., 2012), also contained lower counts of TA genes in our study. Moreover, strain CC1557 that was shown to contain unusually lower counts of type III effectors in a previous study (Hockett et al., 2014) was predicted to encode fewer of TA system genes (8 TA systems encoded compared to a median of 15). It could be possible that the TA systems occur in same genomic islands as the virulence and resistant determinants and could prevent their loss, imparting adaptational benefits. Alternatively, these results might indicate that strains that rely on larger repertoires of type III effectors, also rely on larger TA system repertoires in a manner related to their ecology. Testing these alternative hypotheses will require a combination of additional bioinformatic analyses and experimentation.

Taken together, we show that *P. syringae* genomes encode multiple TA systems that target diverse cellular functions upon activation. Based on our results of the abundance patterns and evidence of the ecological roles discussed above, we speculate that TA systems could be important as virulence effectors, in maintaining genes of virulence and antimicrobial resistance, in antimicrobial and environmental stress tolerance, in defending phage predation among others in *P. syrinage*. Further research focusing on the experimental characterization and ecological roles of the predicted TA systems can provide important insights in the epidemiology and management of plant disease caused by *P. syringae*.

## Data Availability Statement

The original contributions presented in the study are included in the article/Supplementary Material, further inquiries can be directed to the corresponding author/s.

## Author Contributions

PK, LT, and KH: conceptualization and writing manuscript drafts. PK, MN, CF, and RP: data acquisition and analysis. LT and KH: supervision and funding acquisition. All authors: revision of drafts.

## Conflict of Interest

The authors declare that the research was conducted in the absence of any commercial or financial relationships that could be construed as a potential conflict of interest.

## Publisher’s Note

All claims expressed in this article are solely those of the authors and do not necessarily represent those of their affiliated organizations, or those of the publisher, the editors and the reviewers. Any product that may be evaluated in this article, or claim that may be made by its manufacturer, is not guaranteed or endorsed by the publisher.

## Footnotes

1
https://www.kazusa.or.jp/codon/

2
https://katlabs.cc/genegraphics/app

## Supplementary Material

Supplementary Table 1 *Pseudomonas syringae* strain genomes used in this study.

Supplementary Table 2 Sequences and other details on toxin antitoxin systems of the three model strains DC3000, B728a, and 1448A.

Supplementary Table 3 Data on transcriptomics study analyzed for TA systems in this study.
