# Supplementary material for: Genome Mining Shows Ubiquitous Presence and Extensive Diversity of Toxin-Antitoxin Systems in Pseudomonas syringae
Source: Front Microbiol. 2022 Jan 12;12:815911. doi: 10.3389/fmicb.2021.815911 (PMC8790059; doi:10.3389/fmicb.2021.815911)
Supplement: Supplementary file 4 [file Image_1.PDF]

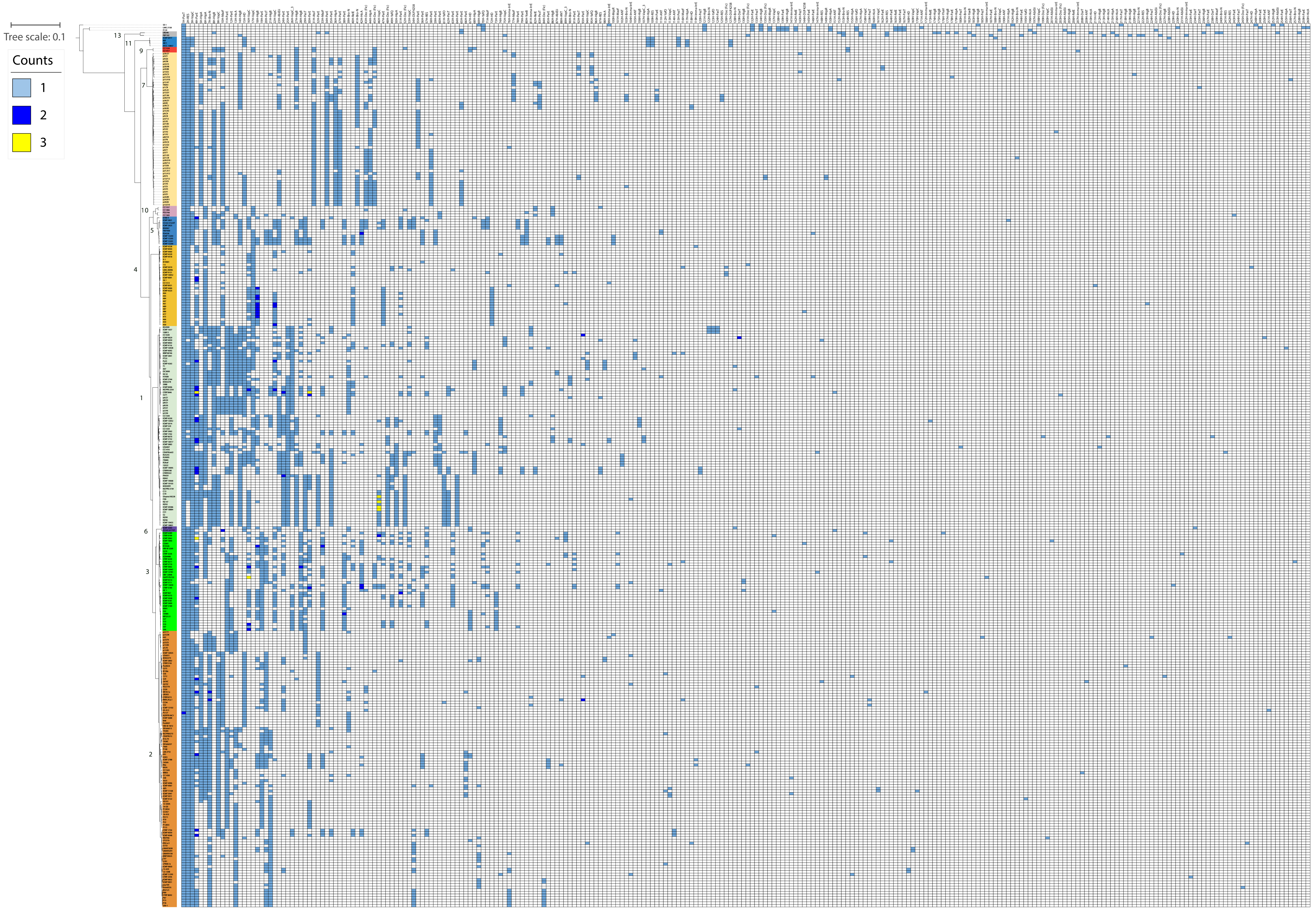

Fig. S1. Heatmap showing abundance of 260 toxin hits predicted in SLING for the 339 P. syringae strains.
