# Supplementary material for: Genome Mining Shows Ubiquitous Presence and Extensive Diversity of Toxin-Antitoxin Systems in Pseudomonas syringae
Source: Front Microbiol. 2022 Jan 12;12:815911. doi: 10.3389/fmicb.2021.815911 (PMC8790059; doi:10.3389/fmicb.2021.815911)
Supplement: Supplementary file 5 [file Image_2.PDF]

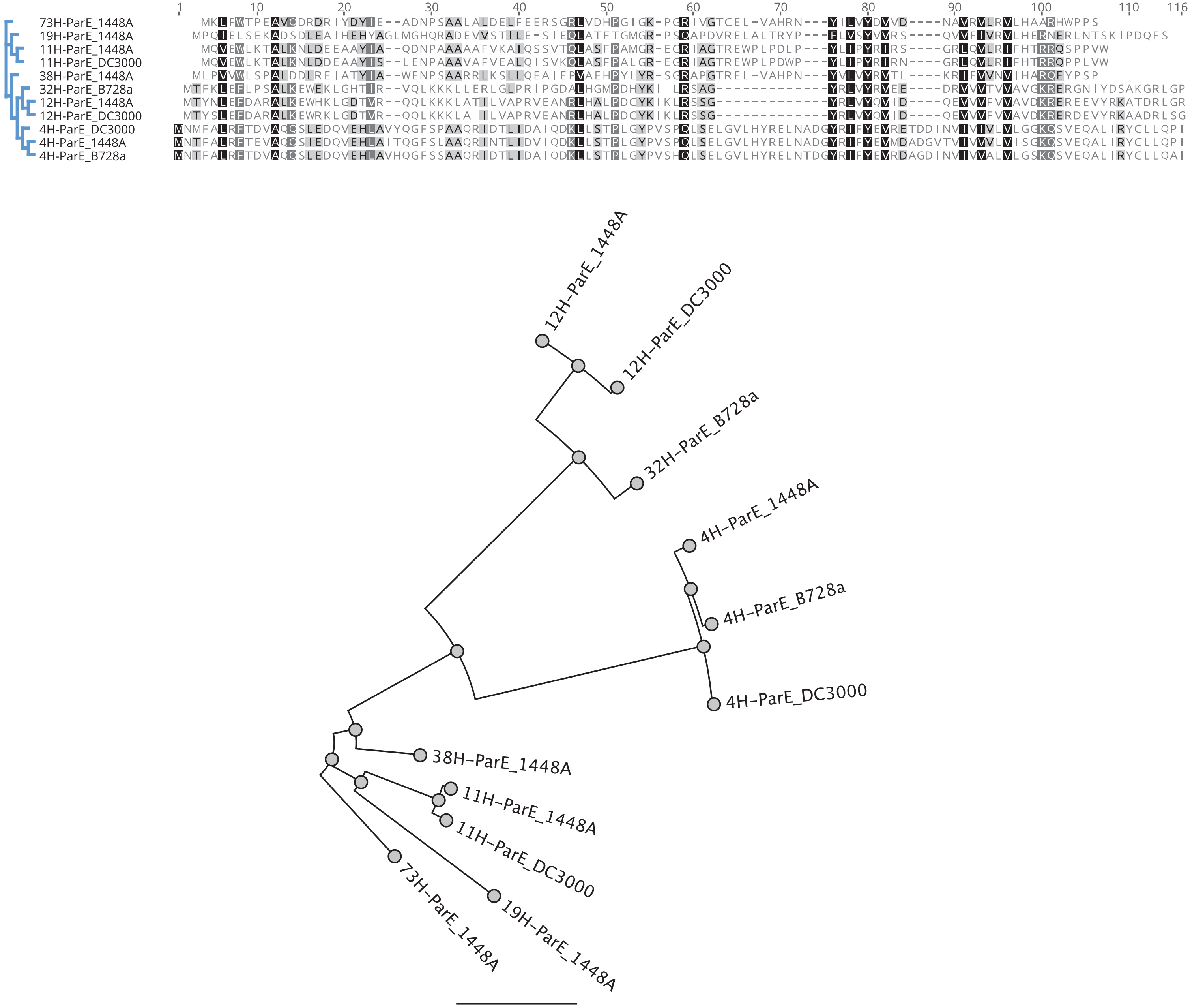

Fig. S2. Alignment (up) and clustering (down) of different parE homologs in the three model strains B728a, 1448A, and DC3000. Orthologs with same hit names were conserved (eg. 4H-parE), but paralogs with different hit names were not conserved (eg. parE of 1448A).
