# Supplementary material for: Genome Mining Shows Ubiquitous Presence and Extensive Diversity of Toxin-Antitoxin Systems in Pseudomonas syringae
Source: Front Microbiol. 2022 Jan 12;12:815911. doi: 10.3389/fmicb.2021.815911 (PMC8790059; doi:10.3389/fmicb.2021.815911)
Supplement: Supplementary file 6 [file Image_3.PDF]

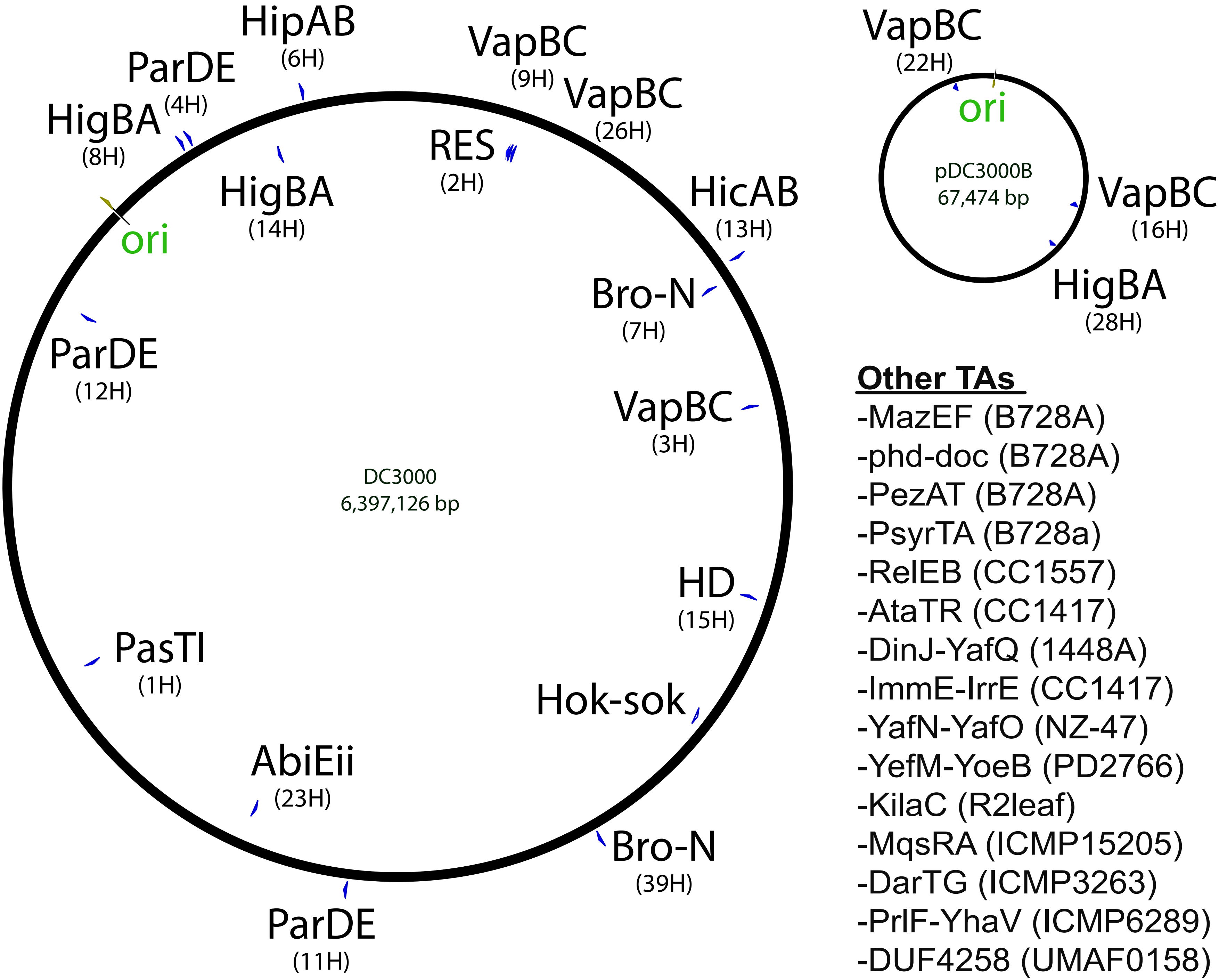

Fig. S3. Genomic map and distribution of predicted TA systems of P. syringae. TA systems in the type strain DC3000 chromosome and plasmid are presented. TA systems not predicted in DC3000 but in other strains are presented as other TAs with a representative strain name attached. Except for the abiEii and hok-sok system, all TA systems belong to the type II class.
