# Supplementary figures and images for: Genome Mining Shows Ubiquitous Presence and Extensive Diversity of Toxin-Antitoxin Systems in Pseudomonas syringae
Source: Front Microbiol. 2022 Jan 12;12:815911. doi: 10.3389/fmicb.2021.815911 (PMC8790059; doi:10.3389/fmicb.2021.815911)

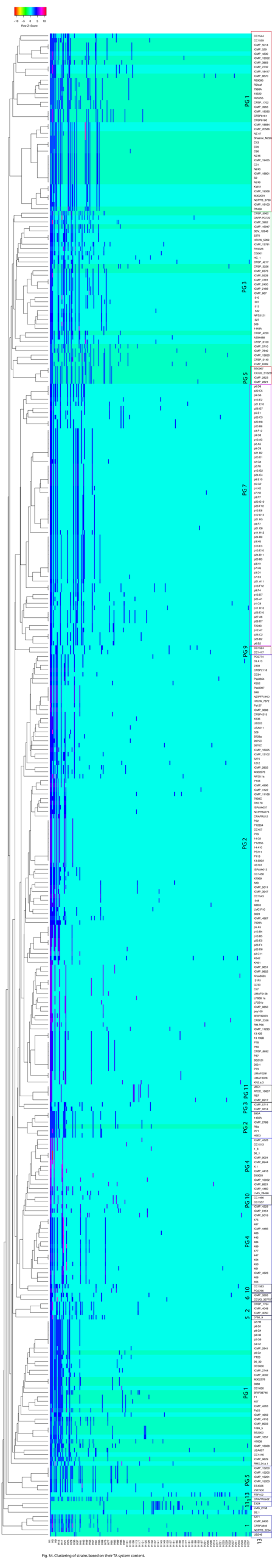

Fig. S4. Clustering of strains based on their TA system content.

Supplement: Supplementary file 7 [file Image_4.PDF]
