# Supplementary material for: Genome Mining Shows Ubiquitous Presence and Extensive Diversity of Toxin-Antitoxin Systems in Pseudomonas syringae
Source: Front Microbiol. 2022 Jan 12;12:815911. doi: 10.3389/fmicb.2021.815911 (PMC8790059; doi:10.3389/fmicb.2021.815911)
Supplement: Supplementary file 8 [file Image_5.PDF]

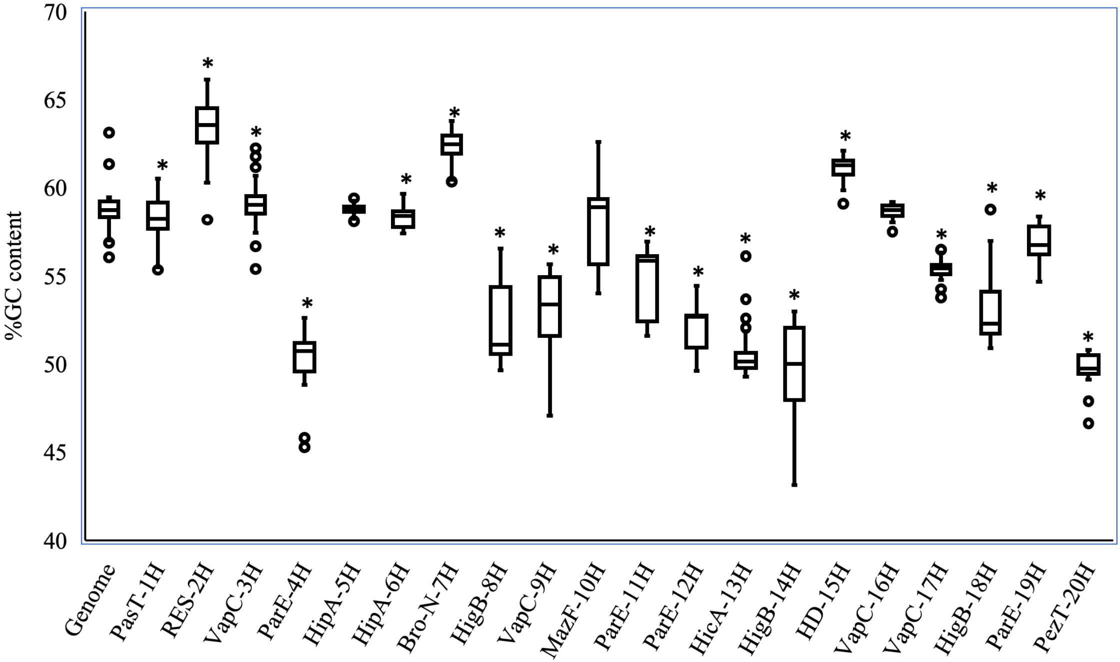

Fig. S5. Variation in GC content of TA systems. GC content of the 339 genomes and the top 20 toxin hits from the strains in which a hit was found are shown. * indicates significance difference in GC content between the toxin gene and genome based on Steel test at P=0.05.
