# Supplementary material for: Genome Mining Shows Ubiquitous Presence and Extensive Diversity of Toxin-Antitoxin Systems in Pseudomonas syringae
Source: Front Microbiol. 2022 Jan 12;12:815911. doi: 10.3389/fmicb.2021.815911 (PMC8790059; doi:10.3389/fmicb.2021.815911)
Supplement: Supplementary file 9 [file Image_6.PDF]

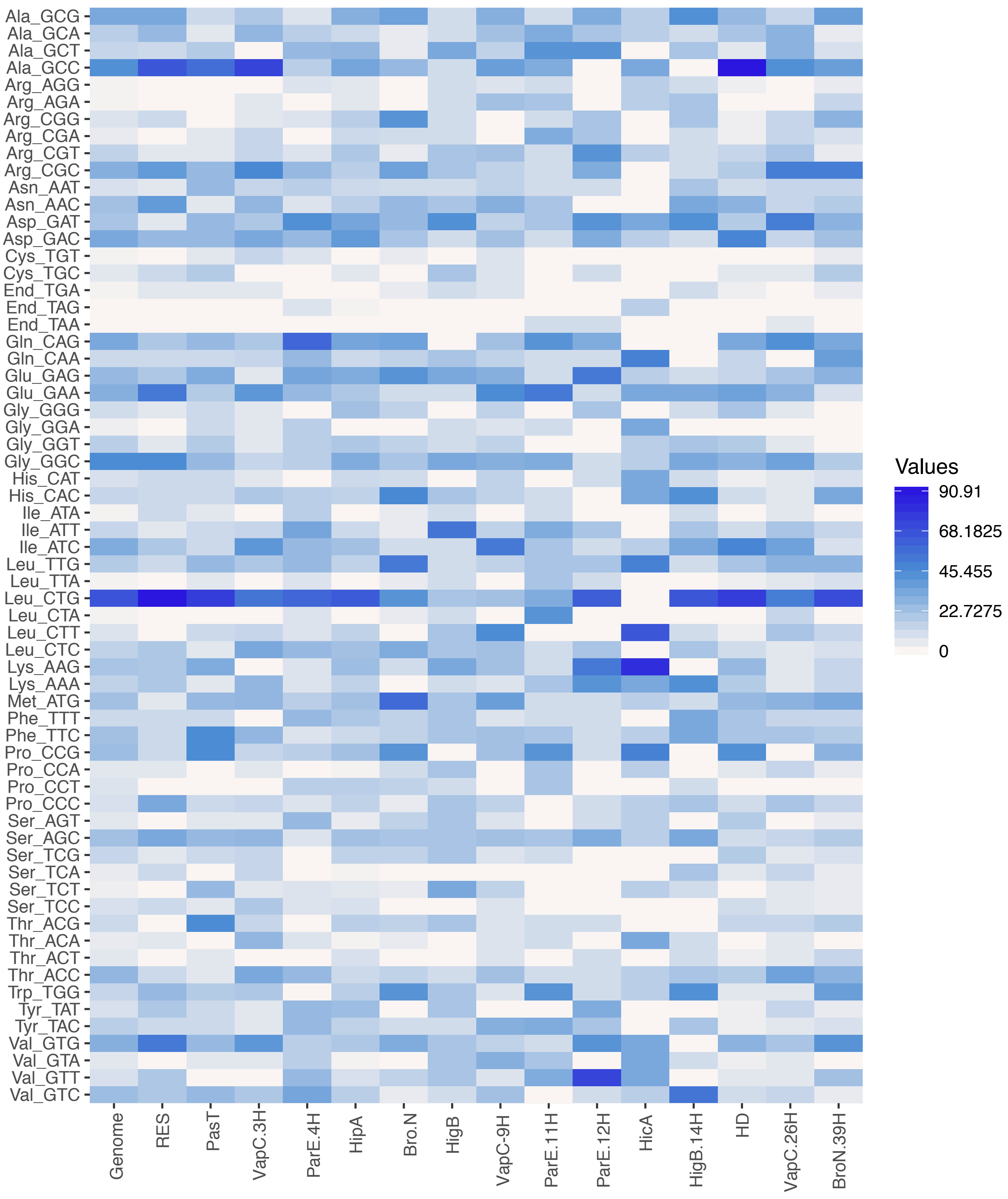

Fig. S6. Variation in codon usage of TA toxins in comparison to genome for strain DC300. Heatmap shows the codon use frequency of each of the codon per 1000 codons
