# Supplementary material for: Genome Mining Shows Ubiquitous Presence and Extensive Diversity of Toxin-Antitoxin Systems in Pseudomonas syringae
Source: Front Microbiol. 2022 Jan 12;12:815911. doi: 10.3389/fmicb.2021.815911 (PMC8790059; doi:10.3389/fmicb.2021.815911)
Supplement: Supplementary file 10 [file Image_7.PDF]

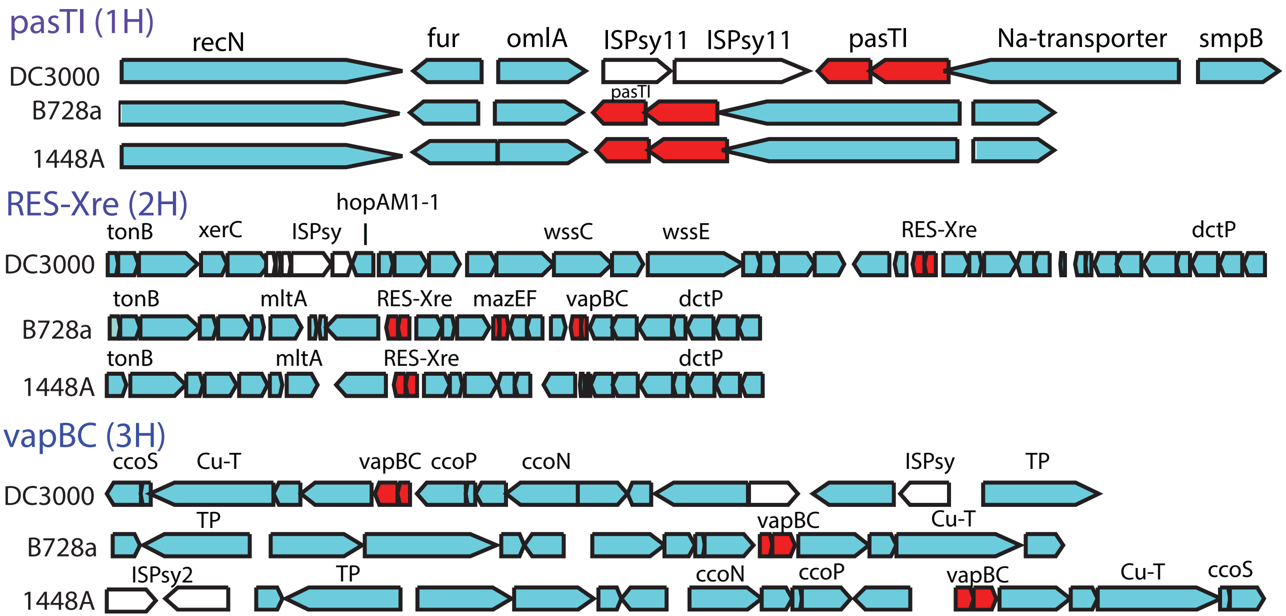

Fig. S7. Gene neighborhood analysis of the three major chromosomally linked TA systems. All three were associated to some mobile elements at least in one of the three model strains examined. (Gene color code; red-TA genes, white-transposon or IS element gene, cyan-other genes). In DC3000, RES was associated to a type III effector gene and the wss operon genes and vapBC region was inverted.
