# Supplementary material for: Genome Mining Shows Ubiquitous Presence and Extensive Diversity of Toxin-Antitoxin Systems in Pseudomonas syringae
Source: Front Microbiol. 2022 Jan 12;12:815911. doi: 10.3389/fmicb.2021.815911 (PMC8790059; doi:10.3389/fmicb.2021.815911)
Supplement: Supplementary file 11 [file Image_8.PDF]

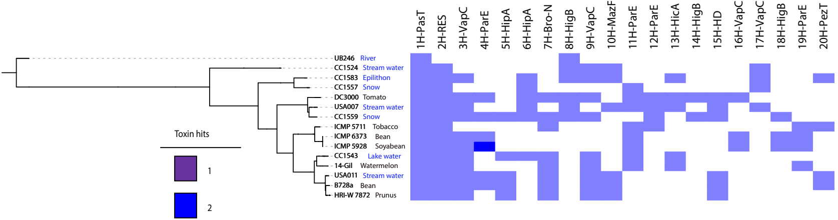

Fig. S8. Heatmap of Top 20 hits of TA toxins by SLING. Isolates from sources other than plant are indicated with blue label in the strain name.
