# Supplementary material for: Genome Mining Shows Ubiquitous Presence and Extensive Diversity of Toxin-Antitoxin Systems in Pseudomonas syringae
Source: Front Microbiol. 2022 Jan 12;12:815911. doi: 10.3389/fmicb.2021.815911 (PMC8790059; doi:10.3389/fmicb.2021.815911)
Supplement: Supplementary file 12 [file Image_9.PDF]

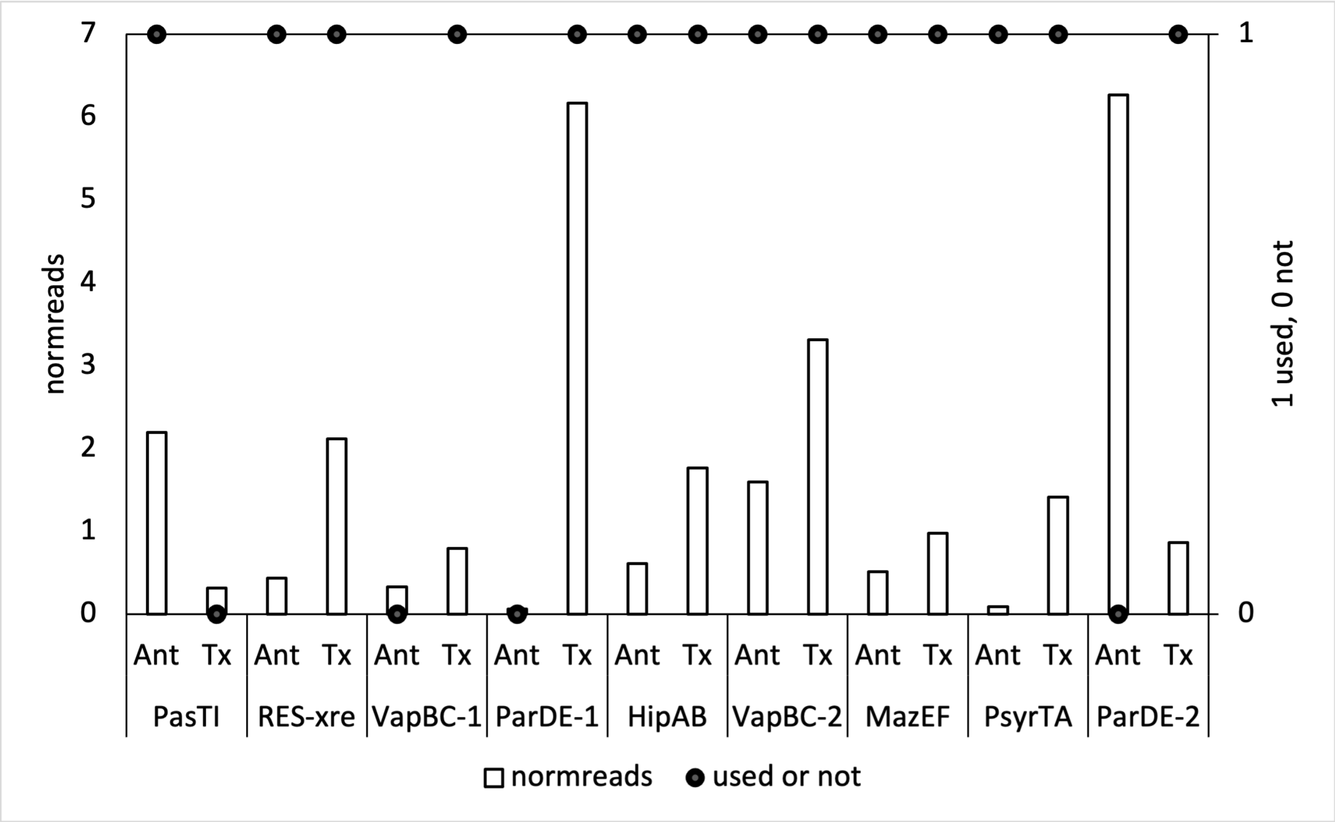

Fig. S9. Fitness scores of toxins (Tx) and antitoxin (Ant) genes of predicted TA systems in the strain B728a. Y-axis on the left shows number of reads that lie within the gene and normalized so that the median across all genes is 1, and also normalized for GC content. Y-axis on the right shows if the gene contained enough reads to be used in experiments. Genes that were not used were important for in vitro growth
